# Absence of p300 induces cellular phenotypic changes characteristic of epithelial to mesenchyme transition

**DOI:** 10.1038/sj.bjc.6603101

**Published:** 2006-04-18

**Authors:** D Krubasik, N G Iyer, W R English, A A Ahmed, M Vias, C Roskelley, J D Brenton, C Caldas, G Murphy

**Affiliations:** 1 Department of Oncology, University of Cambridge, Cambridge Institute for Medical Research, Addenbrooke's Hospital, Hills Road, Cambridge CB2 2XY, UK; 2 Cancer Genomics Program, Department of Oncology, University of Cambridge, Hutchison/MRC Research Centre, Hills Road, Cambridge CB2 2XZ, UK; 3 Department of Anatomy, University of British Columbia, 2177 Westbrook Mall, Vancouver BC V66T 1Z3, UK

**Keywords:** p300, HCT116, homologous recombination, E-cadherin

## Abstract

p300 is a transcriptional cofactor and prototype histone acetyltransferase involved in regulating multiple cellular processes. We generated p300 deficient (p300^−^) cells from the colon carcinoma cell line HCT116 by gene targeting. Comparison of epithelial and mesenchymal proteins in p300^−^ with parental HCT116 cells showed that a number of genes involved in cell and extracellular matrix interactions, typical of ‘epithelial to mesenchyme transition’ were differentially regulated at both the RNA and protein level. p300^−^ cells were found to have aggressive ‘cancer’ phenotypes, with loss of cell–cell adhesion, defects in cell–matrix adhesion and increased migration through collagen and matrigel. Although migration was shown to be metalloproteinase mediated, these cells actually showed a downregulation or no change in the level of key metalloproteinases, indicating that changes in cellular adhesion properties can be critical for cellular mobility.

p300 was initially cloned as an adenoviral E1A-binding protein and subsequently characterised as a transcriptional coactivator with histone acetyl transferase activity. It promotes gene transcription by bridging DNA-binding transcription factors and the basal transcription machinery, by providing a scaffold to integrate transcription factors and by modifying the activity of transcription factors and chromatin through direct acetylation of specific lysine residues. p300 and its close homologue CREB-binding protein (CBP) play a key role in a diverse array of cellular processes including cell cycle regulation, proliferation, differentiation, apoptosis, DNA damage repair and adhesion, as well as embryonic development (Chan and La Thangue, 2001; Iyer *et al*, 2004b). Mutations in p300/CBP have been found in a number of human cancers. Bi-allelic somatic mutations in the p300 gene have been identified in gastric, colon and breast cancers (primary tumours and cancer cell lines), and some of these mutations clearly result in inactivated or truncated protein products (Muraoka *et al*, 1996; Gayther *et al*, 2000; Ozdag *et al*, 2002). While this gave rise to the view that p300 could function as a classical tumour suppressor, it is still unclear how a loss of p300 and CBP could contribute to tumorigenesis (Goodman and Smolik, 2000).

It has been recently proposed that a major process contributing to tumour progression is epithelial to mesenchyme transition (EMT) (Thiery, 2002). Epithelial to mesenchyme transition describes the de-differentiation of polarised epithelial cells to mesenchymal cells, which characteristically have a more motile phenotype. Such a loss of epithelial characteristics is often found in carcinomas with a greater malignant potential and EMT is believed to occur during the transition of adenomas to adenocarcinomas in colorectal cancer (Thiery, 2002). Extracellular matrix (ECM) degrading proteases (including serine-, metallo- and cysteine proteases) play a key role in the invasion of tumour cells, and these could function as the mediators of the EMT-induced invasiveness.

In this study, we used a somatic cell line knockout model to study the cancer phenotype induced by disrupting p300 (Iyer *et al*, 2004a). Loss of p300 in HCT116 cells results in a potentially aggressive phenotype characterised by reduced adhesion and increased migration. In addition, several critical genes involved in these pathways are differentially expressed, suggesting that p300 loss promotes EMT.

## MATERIALS AND METHODS

### Cell culture, generation of p300^−^ and rescue cells

HCT116 (ATCC, Manassas, VA, USA) and its derivatives were cultured in McCoy's 5A medium with 10% fetal calf serum (FCS) and penicillin/streptomycin (Invitrogen, Paisley, UK). p300 gene targeting was performed as previously described (Iyer *et al*, 2004a). HCT116 cells were chosen because they are amenable to homologous gene recombination and p300 has been found to be mutated in many colorectal cancers. Briefly, the single expressed p300 allelle in HCT116 was targeted by homologous recombination resulting in cells null for any expressed p300 protein. Three separately targeted clones were derived and used in each experiment. Rescue clones were made by co-transfecting pcDEF-Flag-p300 (kind gift from MA Ikeda) (Suganuma *et al*, 2002), with pPGK-Puro at a 10 : 1 ratio, and selecting with 1 *μ*g ml^−1^ Puromycin (Sigma, Cambridge, UK) for 24 h. Two separate EF-wt rescue clones were derived and used in each experiment. p300 expression levels in each clone were determined at the start of every experiment. Rescue cells were generated using a p300 expression construct under the control of an EF-promoter (human elongation factor 1*α* promoter). This was preferred as it resulted in stable expression over longer time periods (Suganuma *et al*, 2002).

### Immunofluorescence localisation

Cells were cultured on cover slips, fixed with 4% paraformaldehyde (PFA) solution, blocked with 1 mM glycine (pH 7.5) and permeabilised for 4 min in 0.1% Triton. Immunofluorescent detection was carried out in duplicate (Roghi and Allan, 1999). The primary antibodies used were: E-cadherin (BC Biosciences, San Jose, CA, USA, 1 : 50), ZO-1 (Invitrogen, Zymed, Paisley, UK, 1 : 100), *β*-catenin (Transduction Labs, 1 : 50), Vimentin (DAKO, 1 : 50). The AlexaFluor 488 conjugated anti-mouse antibody (Invitrogen, Molecular Probes, Paisley, UK, 1 : 500) was the secondary antibody in all cases. Pictures were taken with a × 63 objective.

### Western blots

Cells were harvested in protein lysis buffer (50 mM Tris/HCl, 150 mM NaCl, 1% NP-40 v v^−1^, 10 mM EDTA, Complete™ Inhibitor Cocktail 1 tablet 10 ml^−1^ (Roche Diagnostics, Welwyn Garden City, UK)). Samples were subjected to reducing SDS–PAGE and transferred to nitrocellulose using a semidry blotter. The membranes were blocked in 5% fat-free milk and subsequently incubated in the primary and secondary antibody solution for 1 h each. Blots were visualised using the Enhanced chemi-luminescence detection system from Amersham. All experiments and blots were performed in triplicate and representative examples are shown in the figures. Cytosolic and nuclear fractions were obtained with the BioVision Nuclear/Cytosol Fractionation Kit. Membrane fractionation was performed as previously described (Smith *et al*, 2005). Primary antibodies were mouse monoclonal E-cadherin (Zymed; 1 : 500), Vimentin (DAKO; 1 : 250), ZO-1(Zymed; 1 : 500), *α*-tubulin (B-152; 1 : 5000), the polyclonal rabbit anti-p300 (Santa Cruz Biotechnology, Santa Cruz, CA, USA, 1 : 200) and anti-*β*-actin (ab8227; 1 : 5000). The secondary antibody was a sheep anti-mouse or a donkey anti-rabbit horseradish peroxidase conjugated monoclonal antibody (Jackson Immuno Research Europe, Soham, UK, 1 : 5000).

### Matrigel growth

For three-dimensional (3-D) cultures, cells were plated at a density of 1 × 10^5^ cm^−2^ onto reconstituted (pre-gelled) basement membrane (Matrigel; BD Biosciences, San Jose, CA, USA) in DMEM/F12 media with 2% FCS. Cells were incubated for 18 h and photographed live by phase microscopy. For quantification of cluster sizes, cells were subsequently fixed in 4% PFA in PBS for 10 min and nuclei stained with 0.5 *μ*g *μ*l^−1^ DAPI for 5 min. Cells were mounted, and cluster sizes were determined by counting nuclei using a Zeiss Axiphot microscope equipped for epifluorescence. For immunoflurescence, 3-D basement membrane cultures of cells were maintained on glass coverslips and grown to subconfluence. Cells were fixed with methanol for 15 min, rinsed three times with PBS and blocked in PBS with 1% bovine serum albumin (BSA).

### Determination of integrin cell surface levels

Cell surface levels of integrins were quantified by flow cytometry. Cells, 2.5 × 10^5^,were detached using 5 mM EDTA in PBS, centrifuged at 200 × **g** for 4 min and washed in ice-cold PBS three times. All reagents used from this stage on were precooled on ice. Cells were incubated in 100 *μ*l PBS containing 0.5% BSA and the primary antibody at 10 *μ*g ml^−1^ for 1 h on ice (integrin *α*_2_ was purchased from Dianova, integrin *α*_1_ from Serotec, integrin *α*_5_ from DAKO, integrin *α*_6_ from BD Pharmingen, integrin *α*_V_ from Chemicon, integrin *β*_1_ from BD Pharmingen, integrin *β*_3_ from Serotec). Cells were washed in PBS/BSA and incubated in 100 *μ*l PBS/BSA solution containing the secondary antibody (AlexaFluor 488 goat anti-mouse, AlexaFluor 488 goat anti-rat 1 : 500 in the PBS/BSA solution). The cells were fixed in 2% PFA for 20 min and washed once in PBS. Experiments were conducted in duplicate.

### Adhesion assay

Black immunotreated 96-well plates (Nunc maxisorb) were coated overnight with the matrix components in coating buffer at 10 *μ*g ml^−1^ and blocked for 2 h with 100 *μ*l coating buffer/1% BSA (Sigma Aldrich, Gillingham, UK) solution. As negative control wells were blocked with the BSA solution only. Cells were removed from their culture vessels with 5 mM EDTA in PBS and were dye loaded with 2 *μ*M CMFDA cell tracker dye (Molecular Probes) according to the manufacturer's protocol. 10^5^ cells were incubated in HBSS with 1 mM Mn^2+^ at 37°C for 1.5 h in the coated wells. All samples were analysed in quadruplicate. After incubation the fluorescence was measured in a plate reader (Tecan Spectrafluor Plus, excitation 485 nm, emission 595 nm). The wells were washed 5 × with 200 *μ*l HBSS. The fluorescent values of each sample were averaged and the mean background value was subtracted. To inhibit the adhesion mediated by integrin *α*_2_ an integrin *α*_2_ binding antibody (Dianova, A.1.43) was added during the adhesion phase of the assay. The antibody was used at concentrations between 1 *μ*g ml^−1^ and 30 *μ*g ml^−1^ for the inhibition of adhesion.

### Boyden chamber migration assay

The relative migration of wild-type (WT) and p300^−^ cells was studied with an *in vitro* migration assay, conducted in Boyden chambers (Becton Dickinson, 8 *μ*m pores). The membrane of the cell culture insert was coated with 100 *μ*l collagen I (100 *μ*g ml^−1^, Sigma) or growth factor-reduced matrigel (200 *μ*l ml^−1^, BD Biosciences, San Jose, CA, USA) in coating buffer (0.1 M NaHCO_3_ (pH 9.8)) for 16 h. WT and p300^−^ HCT116 cells were dye loaded with 2 *μ*M CMFDA cell tracker dye (Molecular Probes). Cells, 2.5 × 10^5^, were added to the cell culture insert and incubated for 22 h. Membranes were then fixed for 5 min in methanol at −20°C, mounted on a slide and 15 randomly chosen fields of view were photographed at 40 × magnification. Cells were counted, averaged over the three membranes and expressed in relation to the WT.

### cDNA Synthesis and qPCR

RNA was isolated with the Promega kit according to the manufacturers protocol. In all, 1 *μ*g of total RNA was reverse transcribed using 2 *μ*g random hexamers (Amersham Biosciences, Amersham, UK) and 200 Units of Superscript II reverse transcriptase (Invitrogen Life Technologies, Paisley, UK), according to the supplier's instructions. Quantitative polymerase chain reaction (PCR) reactions were performed on a ABI Prism 7700 (Applied Biosystems, Warrington, UK; (Nuttall *et al*, 2003), with each reaction containing 5 ng of reverse-transcribed RNA. Primers and fluorogenic probes for a selection of matrix metalloproteinase (MMPs) and a disintegrin and metalloproteinase (ADAMs) were obtained by courtesy of D Edwards, UEA, Norwich (Koshy *et al*, 2002; Nuttall *et al*, 2003).

## RESULTS

### Comparison of HCT116 and p300^−^ cells reveals an expression profile associated with EMT and a disruption in cell-cell adhesion

**Figure 1:**
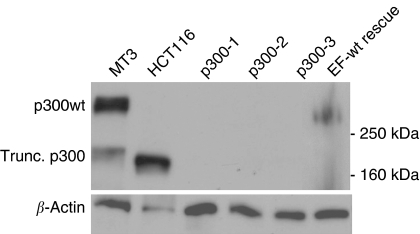
Western blot of p300. Immunoblots showing p300 levels in HCT116 WT, three independently targeted p300^−^ clones (p300-1, p300-2, p300-3) and two EF-wt rescue clones, where p300 was reintroduced, with *β*-actin loading control. MT3 cells express the full-length p300 construct that was introduced in the EF-wt rescue clones, whereas HCT116 express an truncated form. The experiment was repeated three times and a representative blot is displayed.

HCT116 derivatives null for p300 (p300^−^ cells) were generated as previously described (Iyer *et al*, 2004a), and three independently targeted p300^−^ clones (p300^−^1, p300^−^2, p300^−^3) were used for all subsequent experiments. p300 expression was tested by Western blot (Figure 1). To characterise the effect of p300 loss on gene expression, cDNA microarray profiling was initially performed using the 6 K genomewide cDNA microarrays (from ICR, Sutton, UK) as previously described (Ahmed *et al*, 2004). Data from these experiments showed that p300^−^ cells have a gene expression profile characteristic of EMT. These include a reduced expression of several members of the cadherin and integrin families and CD44. Downregulation of E-cadherin and upregulation of Vimentin was observed in two of the three clones, but overall neither were statistically significant (G Iyer and C Caldas, unpublished).

**Figure 2:**
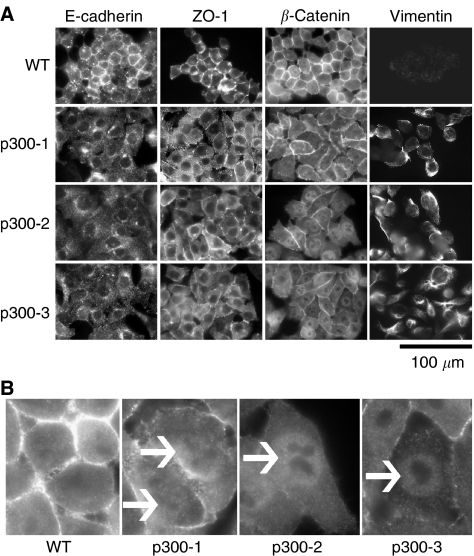
Immunofluorescent localisation of epithelial and mesenchymal markers. Cells were stained for the epithelial markers E-cadherin, ZO-1, *β*-catenin and the mesenchymal marker Vimentin (**A**). Experiments were repeated three times; one representative field of view is represented for each cell type. (**B**) Shows a magnified version of the nuclear staining of *β*-catenin in the WT compared to the p300^−^ cells.

**Figure 3:**
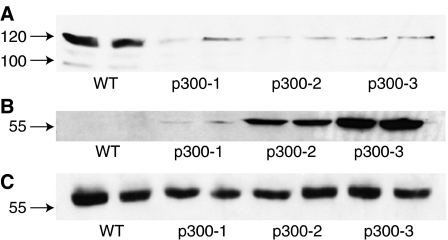
Western blots of (**A**) E-cadherin and (**B**) Vimentin. Total cell lysates were analysed in duplicate from independent wells. Monoclonal mouse primary antibodies and a sheep anti-mouse horseradish peroxidase-conjugated secondary antibody were used for detection. (**C**) shows staining *α*-tubulin on the same membrane as a loading control. The experiments were repeated three times and a representative blot is displayed.

**Figure 4:**
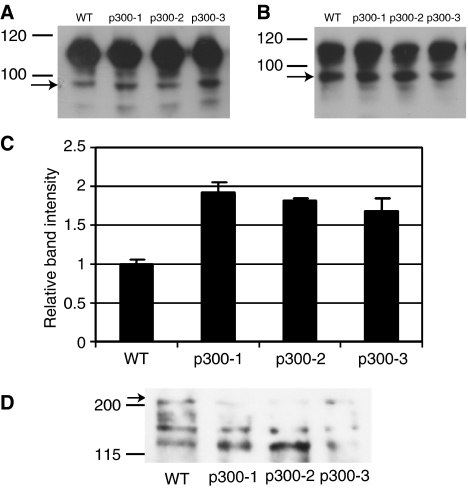
Western blots of (**A**) nuclear, (**B**) cytosolic extracts, (**C**) statistical analysis of *β*-catenin and (**D**) Western blot of the membrane fractionation for ZO-1. Nuclear and cytosolic extracts were prepared from WT and p300^−^ cells and analysed by Western blot. The *β*-catenin band at 92 kDa is indicated by an arrow. The band at 110 kDa is nonspecific and serves well as internal standard. Experiments were repeated three times and densitometry and statistical analysis of the three separate experiments (in **C**) showed a significant increase of *β*-catenin in the p300^−^ cells (*P*-value <0.05). (**D**) Shows a blot of ZO-1 in membrane fractions of WT and p300^−^ cells. The band at 225 kDa indicated by an arrow corresponds to ZO-1. The experiment was repeated three times and a representative blot is shown.

We therefore carried out an analysis of key adhesion molecules using more reliable techniques. Immunostaining showed that E-cadherin levels at adherens junctions were significantly lower in p300^−^ cells (Figure 2A). Reverse transcription–polymerase chain reaction (RT–PCR) analyses (data not shown) and Western blot (Figure 3A) confirmed reduced transcription of the E-cadherin gene and reduced protein levels. *β*-Catenin and ZO-1 were found to be redistributed in p300^−^ cells (Figure 2A): decreased staining of *β*-catenin and ZO-1 at cell–cell junctions and increased nuclear *β*-catenin staining (Figure 2B). The increase in nuclear *β*-catenin levels was verified by cellular fractionation and Western blot (Figure 4A), while no change in cytosolic *β*-catenin was observed (Figure 4B). Densitometry of three separate experiments showed that the increase in nuclear *β*-catenin was statistically significant (*P*-value <0.05, Figure 4B). Membrane fractionation confirmed the reduced levels of ZO-1 localising to the membrane in the p300^−^ cells in comparison to the WT cells (Figure 4C). Total cellular levels of both proteins, determined by Western blots, remained unchanged (data not shown). Immunostaining (Figure 2A) and Western blots (Figure 3B) for the mesenchymal intermediate filament Vimentin, showed that all three p300^−^ clones expressed significant amounts of the mesenchymal marker Vimentin (albeit with varying degrees of heterogeneity), while levels were undetectable in HCT116 cells. These results suggest that loss of p300 is associated with changes characteristic of EMT in this colorectal cancer cell line.

### Loss of cell–cell junctions coincides with reduced clustering in matrigel

**Figure 5:**
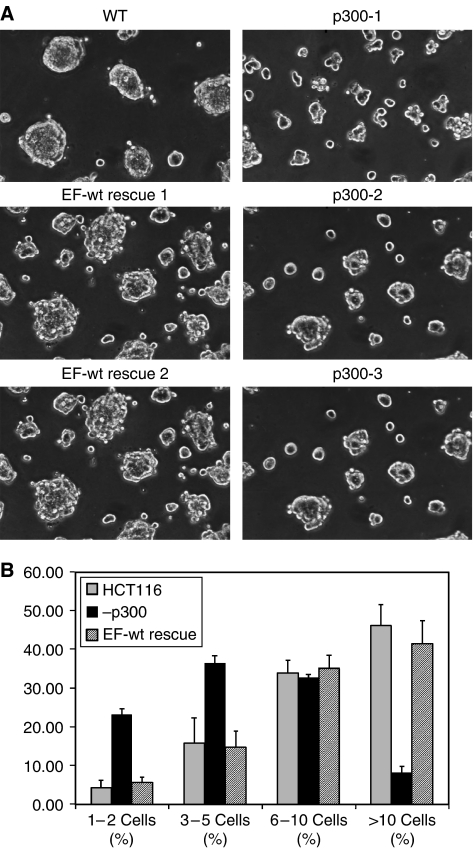
Adhesion defect on Matrigel growth after 18 h. (**A**) Matrigel growth of WT, p300^−^ and EF-wt rescue cell clusters. p300^−^ clones (p300-1, p300-2 and p300-3) cluster poorly into small clusters with ragged edges compared to WT cells. This defect is reversed in EF-wt rescue clones. Experiments were performed in triplicate, and the figure above shows a representative example. (**B**) Graph of cluster size distribution in HCT116, p300^−^ and EF-wt rescue cells. The graph demonstrates the distribution of cluster sizes in HCT116 *vs* the p300- and EF-wt rescue clones. Experiments were performed in triplicate in all three p300- clones (p300-1. p300-2 and p300-3) and two EF-wt rescue clones (EF-wt rescue 1 and EF-wt rescue 2). Error bars indicate one standard deviation.

To identify abnormalities in cell–cell adhesion, cells were grown in reconstituted basement membrane (matrigel) based 3-D cultures. Under these conditions, p300^−^ cells grew in small, loosely adherent clusters with ragged edges characteristic of cells with defects in cell–cell adhesion compared to parental HCT116 (Figure 5A). Reintroduction of p300 in ET-wt rescue clones could reverse this phenotype. Expression of p300 in the rescue clones was confirmed by Western blot (Figure 1). Quantification of this defect confirmed that WT and ET-wt rescue clones indeed formed larger cell clusters than p300^−^ cells (Figure 5B). The difference between p300^−^ clones and WT or ET-wt rescue clones was significant for clusters up to five cells (*P*-value <0.05) as well as for clusters above 10 cells (*P*-value <0.005).

### p300^−^ cells have reduced levels of *α*_2_-integrin and reduced adhesion to collagen-I and IV

**Figure 6:**
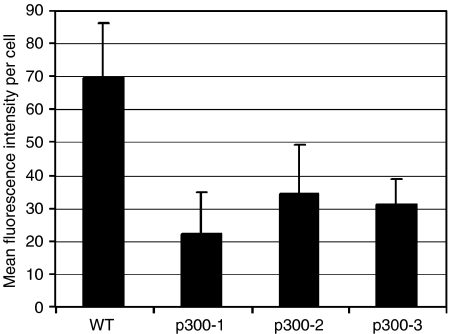
Comparison of integrin *α*_2_ cell surface levels in WT and p300^−^ cells. The samples were measured in duplicate by flow cytometry. The background fluorescence of the secondary antibody was subtracted from the values. The experiment was repeated three times.

**Figure 7:**
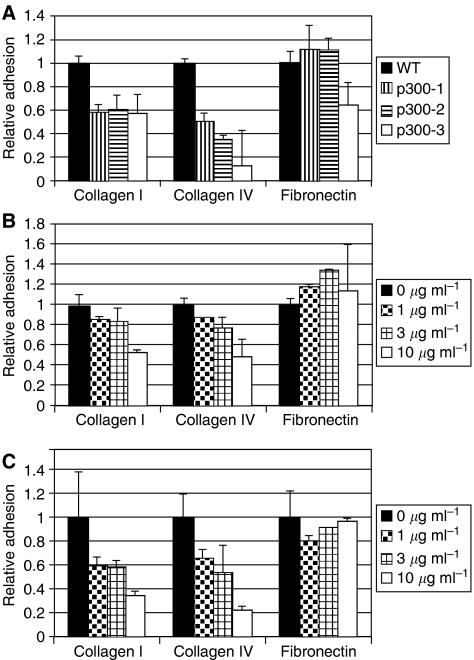
Adhesion of WT and p300^−^ cells. (**A**) Adhesion to different ECM components. (**B** and **C**) show the inhibition of integrin *α*_2_-mediated adhesion in the WT (**B**) and p300^−^ cells (**C**). The concentrations in the legend indicate the concentration of the adhesion blocking integrin *α*_2_ antibody present. Samples were measured in triplicate and experiments repeated three times.

To assess whether the absence of p300^−^ results in abnormal cellular expression of integrins, flow cytometry analyses of surface integrins were performed. These experiments showed a decrease in *α*_2_-integrin (Figure 6), with no significant change in *α*_1_-, *α*_5_-, *α*_V_-, *α*_6_-, *β*_1_- and *β*_3_-integrin levels (data not shown). Major ligands of *α*_2_*β*_1_-integrin are type-I and IV collagen. Therefore, we tested if reduced integrin *α*_2_ levels affected cell–matrix adhesion in p300^−^ cells. *In vitro* adhesion assays showed that p300^−^ cells adhered less to collagen I and matrigel, compared to HCT116 cells (*P*-value <0.05). In contrast, no significant difference in adhesion could be detected for fibronectin (*P*-value >0.05), which is not a ligand of integrin *α*_2_ (Figure 7A). Inhibition of integrin *α*_2_-mediated adhesion by addition of an integrin *α*_2_ blocking antibody reduced adhesion of WT and p300^−^ cells to collagen I and matrigel significantly (for the highest antibody concentration *P*<0.05), however, as expected not the adhesion to fibronectin (Figure 7B and C).

These results show that HCT116 cells rely primarily on *α*_2_-integrins for adhesion to collagen I and IV and suggest that the decrease in cell surface integrin *α*_2_ levels seen in p300^−^ cells results in reduced adhesion.

### p300^−^ cells show increased migration through collagen-I and matrigel-coated membranes

**Figure 8:**
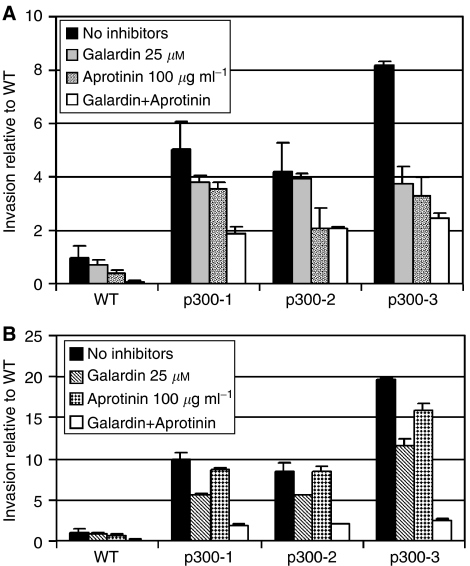
Migration through matrigel (**A**) and collagen-I (**B**) coated membranes in a chemotractive FCS gradient. Experiments were conducted in triplicate and repeated three times. Where indicated metalloproteinase- (Galardin at 25 *μ*M) or the serine protease inhibitor (aprotinin at 100 *μ*M) were added to the medium in the well and the insert.

Defects in adhesion are frequently associated with increased ECM invasion and migration in cancer cells. To compare the motility of HCT116 and p300^−^ cells we used a transwell assay with type-I collagen or matrigel-coated membranes. Migration through the membrane was followed for 22 h in response to a chemotactic gradient of FCS. In these assays, p300^−^ cells demonstrated markedly increased migration through both type-I collagen and matrigel when compared to the WT HCT116 cells. Degradation of ECM and cellular invasion is believed to be primarily dependent on the proteolytic activity of metalloproteinase (MP) and serine protease activity, either by direct degradation of the ECM or as part of a protease activation cascade (DeClerck *et al*, 2004). We showed that migration through type-I collagen was more dependent on MP activity than serine protease activity: GM6001 or Galardin (a broad spectrum MP inhibitor) was more efficient in inhibiting migration through type-1 collagen, compared to aprotinin (a general serine proteinase inhibitor) (Figure 8A). The inhibitor solvent DMSO alone did not affect invasion (data not shown). In contrast, migration through matrigel was inhibited to a similar extent by both aprotinin and Galardin (Figure 8B). In both cases the combination of Galardin and aprotinin significantly inhibited cell migration, but could not abrogate it completely. The motility of the cells on plastic in a wounding assay was not affected by the p300 disruption (data not shown).

### Characterisation of MP and TIMP expression in HCT116 and p300^−^ cells

**Figure 9:**
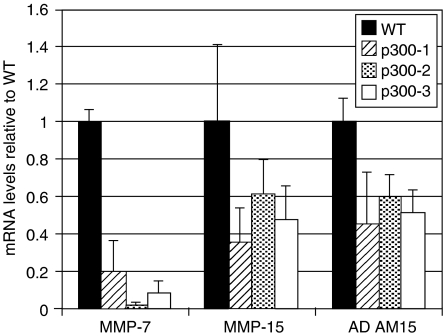
The effect of p300 on the mRNA levels of MMP-7, -15 and ADAM15. MP and TIMP mRNA levels were determined by qRCR in three separate experiments that were conducted in triplicate. The relative expression values were obtained by normalising the *c*_T_-values using the *c*_T_-values of the 18S in the samples as reference, compensated for the exponential fluorescence increase and set in relation to the WT values. *c*_T_-values of MPs in WT: MMP-7: 30; MMP-15: 25; ADAM15: 24.

Our results suggest that increased migration of p300^−^ cells through type-I collagen, could be MP dependent. We therefore proceeded to quantify mRNA levels of known MMP, ADAM, their inhibitors, the tissue inhibitor of metalloproteinase (TIMPs) and members of the serine protease uPA cascade, to assess whether altered expression of these proteins could account for the invasive phenotype observed. Table 1 summarises the results of the real-time RT–PCR analyses. The majority of MPs, TIMP-2 and -3, as well as components of the uPA system were unchanged. However, the mRNA levels of MMP-7, -15 and ADAM15 were significantly decreased in p300^−^ cells (Figure 9). MMP-7 and ADAM15 were found to be below the limits of detection by Western blot (data not shown).

## DISCUSSION

p300 is considered to be a putative tumour-suppressor gene because it is mutated, deleted or underexpressed in a number of cancers. However, the mechanisms, through which p300 functions to suppress tumorigenesis, remain controversial (Chan and La Thangue, 2001; Iyer *et al*, 2004b). Using previously generated p300 deficient clones, derived from a colorectal carcinoma cell line, we propose a novel pathway through which p300 may function as a tumour suppressor.

Disrupting p300 in HCT116 cells induced changes characteristic of EMT, a process thought to be important in carcinogenesis. p300^−^ cells had significant cell–cell adhesion defects, with downregulation of E-cadherin, a major component of the epithelial adherens junctions, and reduced aggregation in matrigel cultures. Reintroduction of p300 could restore the aggregation phenotype. In addition *β*-catenin was found to be increased in nuclear fractions of p300^−^ cells, which may contribute to intracellular signalling inducing EMT. In colorectal tumours, tumour cells at the invasion front display strong nuclear *β*-catenin, and the Tcf4-*β*-catenin transcription factor complex is thought to be involved in EMT (Brabletz *et al*, 2005). p300^−^ cells also showed reduced adhesion to the basement membrane components collagen I and IV consequent to reduced cell surface levels of integrin *α*_2_. The effect of integrin cell surface levels on the invasiveness of cancer cells is debatable and probably depends on the system that is used and relative surface levels and activation status of integrins. Sun *et al* (1998) reported that reexpression of the *α*_2_*β*_1_-integrin in a poorly differentiated breast carcinoma cell line, Mm5MT, resulted in reversion of a malignant phenotype to a differentiated epithelial phenotype. Reduction of integrin *α*_6_*β*_4_ via siRNA in MDA_MB_231 breast cancer cells resulted in decreased invasion on a laminin-coated surface (Lipscomb *et al*, 2003).

Epithelial to mesenchyme transition is often characterised by an increase in invasive potential *in vitro,* and this is believed to foster the invasive and metastatic potential of tumour cells (Thiery, 2002). Comparing the migrative potential of parental HCT116 and p300^−^ cells *in vitro*, we found that p300^−^ cells showed increased migration through collagen I and matrigel-coated membranes. It was not possible to assess migration through a thicker layer of either gel (which would be more representative of cellular invasion), since invasion rates were too low to obtain statistically significant results. Nonetheless, we showed that migration through matrigel was serine- and metalloproteinase dependent, whereas the invasion through collagen I was metalloproteinase and only marginally serine protease dependent.

Interestingly, increased migration was found to be independent of expression levels of proteases involved in collagen or matrigel degradation. Cell surface levels of MMP-14 (determined by flow cytometry) and MMP-2 and MMP-9 activity (determined by zymography) were not affected by the p300 disruption (data not shown). Previous studies have shown that MMP-7 and MMP-14 can be upregulated as a consequence of the movement of *β*-catenin to the nucleus and the increase in the Tcf4-*β*-catenin transcription factor complex. Previous studies have shown that factors other than *β*-catenin can also affect the MMP-7 and MMP-14 promoter activity and the nature of such effectors in this system remain to be elucidated (Crawford *et al*, 2001; Takahashi *et al*, 2002). Other targets of this gene complex include CD44, which was also shown to be downregulated in the microarray analysis of p300^−^ cells. We also noted that MMP-15 was significantly downregulated in p300^−^ cells. Wound healing experiments did not show increased motility of the p300^−^ cells on a plastic culture dish (data not shown). These data indicate that the migration in a chemotactic gradient is supported by the degradation of the ECM coat. However, since protease levels are largely unaffected by the p300 ablation, the increased migration of the p300^−^ cells is most likely due to decreased cell–cell interactions and cell–matrix adhesion. The disruption of cell–cell junctions may allow single p300^−^ cells to migrate more readily through the 8 *μ*m pores of the membrane compared to the clusters of the HCT116 WT cells, as many colon cancer cell lines migrate as cohorts rather than single cells.

The data presented here proposes a novel mechanism through which p300 could function as a tumour suppressor: p300 disruption promotes EMT and an aggressive cancer phenotype. It has been suggested that histone deacetylase (HDAC) inhibitors, which are currently being tested in clinical trials for cancer therapy, function to selectively promote the expression of tumour-suppressor genes. We propose an additional anticancer mechanism, where HDAC inhibitors could promote p300 histone acetylase activity, hence inhibiting EMT and increased invasiveness seen in cancer cells.

## Figures and Tables

**Table 1:** Summary of the effect of p300 ablation on MP, serine protease and TIMP mRNA levels

**MMP**	**Effect of p300 ablation**	**ADAM**	**Effect of p300 ablation**
1	Low expression	8	Low expression
2	Low expression	9	No change
3	No expression	10	No change
7	Downregulation^**^	12	No change
8	No expression	15	Downregulation^**^
9	No expression	17	No change
10	No change	19	No change
11	No change	28	No expression
12	No change	33	No expression
13	No change	TIMP	
14	No change	1	No expression
15	Downregulation^**^	2	No change
16	No expression	3	No change
17	No change	4	No expression
19	No change	Plasmin system	
23	No change	uPAR	No change
25	No change	uPA	No change
		Plasminogen	No expression
		PAI-1	No change
		PAI-2	No change

MP, serine protease and TIMP mRNA levels were determined in triplicate by pPCR and repeated three times. Low expression signifies a *c*_T_-value <35, where quantification becomes inaccurate. Downregulation ^**^corresponds to a *P*-value <0.001 when a mean of the three p300^−^ clones is calculated *vs* the WT.
